# Functionality of *In vitro* Reconstituted Group II Intron RmInt1-Derived Ribonucleoprotein Particles

**DOI:** 10.3389/fmolb.2016.00058

**Published:** 2016-09-27

**Authors:** Maria D. Molina-Sánchez, Fernando M. García-Rodríguez, Nicolás Toro

**Affiliations:** Structure, Dynamics and Function of Rhizobacterial Genomes, Department of Soil Microbiology and Symbiotic Systems, Estación Experimental del Zaidín, Consejo Superior de Investigaciones CientíficasGranada, Spain

**Keywords:** catalytic RNAs, ribonucleoprotein particles, intron-encoded protein, maturase, reverse transcriptase, DNA endonuclease, reverse splicing

## Abstract

The functional unit of mobile group II introns is a ribonucleoprotein particle (RNP) consisting of the intron-encoded protein (IEP) and the excised intron RNA. The IEP has reverse transcriptase activity but also promotes RNA splicing, and the RNA-protein complex triggers site-specific DNA insertion by reverse splicing, in a process called retrohoming. *In vitro* reconstituted ribonucleoprotein complexes from the *Lactococcus lactis* group II intron Ll.LtrB, which produce a double strand break, have recently been studied as a means of developing group II intron-based gene targeting methods for higher organisms. The *Sinorhizobium meliloti* group II intron RmInt1 is an efficient mobile retroelement, the dispersal of which appears to be linked to transient single-stranded DNA during replication. The RmInt1IEP lacks the endonuclease domain (En) and cannot cut the bottom strand to generate the 3′ end to initiate reverse transcription. We used an *Escherichia coli* expression system to produce soluble and active RmInt1 IEP and reconstituted RNPs with purified components *in vitro*. The RNPs generated were functional and reverse-spliced into a single-stranded DNA target. This work constitutes the starting point for the use of group II introns lacking DNA endonuclease domain-derived RNPs for highly specific gene targeting methods.

## Introduction

Group II introns are mobile genetic retroelements present in the genomes of bacteria and organelles. They are related to spliceosomal introns, telomerase and retrotransposons in eukaryotes, and are even considered to have played a key role in the origin of eukaryotic cells and genetic evolution (Koonin, 2006; Lambowitz and Belfort, 2015). In recent years, they have also been used in various biotechnological applications in prokaryotes and eukaryotes, due to their ability to recognize specific sequences for their insertion (Toro et al., 2007; Enyeart et al., 2014). They usually consist of a catalytically active intron RNA and an intron-encoded protein (IEP) that act together as a ribonucleoprotein (RNP) particle (Lambowitz et al., 1999; Saldanha et al., 1999; Lambowitz and Zimmerly, 2004). Immediately after its translation, the IEP is stabilized by binding to the unspliced precursor RNA, facilitating intron lariat excision by successive transesterifications to generate the active RNP complex for intron mobility (recently reviewed in Lambowitz and Belfort, 2015). Thus, the RNP particles can insert the lariat RNA into a short, specific DNA sequence recognized by both RNA and protein components in the reverse reaction to intron splicing (Guo et al., 1997; Singh and Lambowitz, 2001; Jiménez-Zurdo et al., 2003). Some RNP enzymes produce a double strand break (those with an endonuclease domain, see below) and use the 3′ end of the bottom strand to initiate reverse transcription (Zimmerly et al., 1995; Matsuura et al., 1997), but other complexes cannot cut the bottom strand, and an alternative method is therefore required for priming during cDNA synthesis (Muñoz-Adelantado et al., 2003; Martínez-Abarca et al., 2004). The integration process is completed by host enzymes that degrade the RNA, synthesize the complementary strand of cDNA and repair nicks in the DNA (Smith et al., 2005; Coros et al., 2008, 2009; Yao et al., 2013). As described for HIV-1 RT, RNP particles were widely thought to be formed by the interaction of a dimeric protein with a single lariat RNA (Saldanha et al., 1999; Rambo and Doudna, 2004; Gupta et al., 2014). However, a recent determination of the structure of RNP particles obtained *in vivo* suggested that there was a 1:1 (Qu et al., 2016), or even 2:2 (Zhao and Pyle, 2016) ratio of protein and RNA in the complex.

The ribozyme has a conserved, complex three-dimensional structure comprising six domains, DI to DVI (Toor et al., 2008; Qu et al., 2016). All six domains are important for correct structure and catalysis, but domain V plays a key role in nucleating the catalytic residues (the AGC triad and AY bulge) essential for Mg^2+^ coordination and in intron activity (Qin and Pyle, 1998; Gordon and Piccirilli, 2001; Pyle, 2010). The IEP is a multifunctional protein encoded by domain IV in most bacterial introns and about half the known organellar group II introns (Belfort et al., 2002; Lambowitz and Zimmerly, 2011). All these proteins have an N-terminal reverse transcriptase domain (RT) followed by a region involved in RNA splicing (domain X or maturase). Some IEPs also have a DNA binding (D) domain and a C-terminal DNA endonuclease (En) domain. These domains have been extensively characterized in LtrA (the IEP encoded by the *L.lactis* group II intron Ll.LtrB) (San Filippo and Lambowitz, 2002). The RT domain consists of eight (RT 0–7) to twelve (RT 2a, 3a, 4a, and 7a) conserved amino-acid blocks resembling those of telomerase, non-LTR-retrotransposon and retroviral RTs (Blocker et al., 2005; Zhao and Pyle, 2016). Mutational screening and unigenic evolution analyses revealed that the N-terminal RT0 region bound the DIVa subdomain of the ribozyme with high affinity, enabling the protein to regulate its own transcription (Wank et al., 1999; Singh et al., 2002; Cui et al., 2004; Gu et al., 2010). Additional contacts involving other RT and maturase residues seem to be established with the catalytic core of the excised intron RNA (involving DI, DII, and DVI) and are required for the formation of fully functional RNP complexes (Cui et al., 2004; Dai et al., 2008; Qu et al., 2016). The active site of the RT, the YADD motif in RT5, is not required for intron splicing or DNA endonuclease activity (Moran et al., 1995; Cui et al., 2004). By contrast, domain X has been shown to facilitate the excision of the ribozyme in an intron-specific manner (Mohr et al., 1993; Moran et al., 1994; Saldanha et al., 1999). Maturase binding to partially folded introns has been shown to stabilize tertiary interactions in the RNA, leaving other regions relatively flexible for productive conformational changes during catalysis (Matsuura et al., 2001; Noah and Lambowitz, 2003). The D and En domains are required for intron mobility, due to their role in DNA target site binding and cleavage during reverse splicing. The D domain is involved in target DNA recognition, whereas the En domain, an Mg^2+^-dependent DNA endonuclease of the H-N-H family, cleaves the target DNA strand to generate the primer for reverse transcription (San Filippo and Lambowitz, 2002).

However, a large group of introns lack the D/En domains (Toro and Martínez-Abarca, 2013), instead having a short C-terminal extension (Molina-Sánchez et al., 2010). The RTs of these introns thus have no DNA endonuclease activity and cannot cut the bottom strand. RmInt1 is the most widely studied retroelement of this type of group II introns (Martínez-Abarca et al., 2000). Despite lacking the D and En domains, RmInt1 is an efficient mobile element, the dispersal of which appears to be linked to the transient single-stranded DNA formed during replication (Martínez-Abarca et al., 2004). RmInt1 has been shown to self-splice under non-physiological conditions *in vitro* (Costa et al., 2006; Chillón et al., 2014), and to form an intron lariat and circles *in vivo* (Molina-Sánchez et al., 2006). Two mobility pathways, differing in terms of the priming of reverse transcription, have been postulated for RmInt1. The preferred pathway involves the invasion of single-stranded DNA by the intron, with the nascent lagging DNA strand used as the primer for intronic cDNA synthesis. A less efficient alternative pathway has also been suggested, in which the intron probably uses random non-specific opposite strand nicks, a nascent leading strand or the *de novo* initiation of cDNA synthesis.

We show here that the RmInt1 IEP can be readily purified from *Escherichia coli* cells, as a highly soluble fusion protein that is stable and functional in the absence of the intron RNA. The IEP was competent to assist in accurate lariat excision from the precursor intron RNA. Similarly, the intron RNA formed functional RNP particles upon incubation with the purified fusion protein, directing target DNA cleavage and further reverse splicing of the intron lariat into DNA substrates.

## Materials and methods

### Bacterial strains and growth conditions

*Sinorhizobium meliloti* RMO17 was used for mobility assays. It was grown at 28°C on TY or defined minimal medium (Villadas et al., 1995) supplemented with 180 μg·ml^−1^ kanamycin and 10 μg·ml^−1^ tetracycline. *E. coli* DH5α cells were routinely cultured at 37°C in LB medium for molecular cloning. When required, antibiotics were used at the following concentrations: 180 μg·ml^−1^ for kanamycin and 200 μg·ml^−1^ for ampicillin. IEP were expressed in *E. coli* Rosetta-gami (DE3): pLysS (Novagen). This strain was grown at 37°C in LB medium supplemented with 0.2% glucose, 100 μg·ml^−1^ ampicillin, and 50 μg·ml^−1^ chloramphenicol, with protein induction by incubation with 0.3 mM IPTG at 20°C for 16–22 h.

### Plasmid constructs

pMALFlagIEP encodes the IEP tagged with 3xFlag fused to the maltose-binding protein (MBP). This plasmid was constructed by inserting a *Not*I fragment containing the FlagIEP from pCEP4FlagIEP (Reinoso-Colacio et al., 2015) into the pMAL-c5X vector (New England Biolabs) digested with *Not*I and dephosphorylated. pMALFlagIEP mutant constructs were generated by replacing the C-terminal region of the IEP with the corresponding mutated fragment. PCR products containing the various IEP mutations were obtained with Phusion polymerase (Thermo Scientific), the primers 21.0 5′-AGAAAAGACGTC AAATGCAA-3′ and *SacBbr* 5′-GGGAGCTCACGTGCC TCGTTTTCATCGATGAGA-3′, and template plasmids containing the mutated IEP: pKG4-YAHH (Molina-Sánchez et al., 2011); pKG4-K381 and pKG4A354A355 (Molina-Sánchez et al., 2010). These PCR fragments were digested with *Kpn*I and *Pml*I and inserted into the *Kpn*I/*Eco*RV-digested pMALFlagIEP.

pLMWT, which was used for *in vitro* transcription of the intron precursor, was described in a previous study by Chillón et al. (2014) (ΔORF-WT2). It consists of the pUC19 backbone plus15 bp of the RmInt1 5′ exon from IS*Rm2011-2*, the ΔORF intron sequence and 5 bp of the 3′ exon followed by 145 bp of the *E. coli lac*Z gene (positions 42–169). ΔORF is an engineered ribozyme in which nucleotides 611–1759 of the large terminal loop of the RmInt1 domain IV have been deleted (Costa et al., 2006).

The construction of control donor (pKGEMA4) and recipient (pJB0.6LAG) plasmids for *in vivo* retrohoming analysis has been described elsewhere (Martínez-Abarca et al., 2004; Nisa-Martínez et al., 2007). pKG4_MALFlagIEP was generated from the pKGEMA4 donor plasmid by replacing the wild-type IEP sequence with the MBP-FlagIEP fusion protein sequence. The MBP-FlagIEP N-terminal region was amplified from pMALFlagIEP with primers LMS33 5′-GGACTAGTGGAAACAGG **ATG**AAAATCGAAGAAGG-3′ and PR1000 5′-GCGGAA GATTGTCAAACAGC-3′ and Phusion polymerase (Thermo Scientific). LMS33 hybridized to the start of the maltose-binding protein sequence (the ATG start codon is shown in bold) and included a *Spe*I restriction site (underlined), to facilitate cloning in pKGEMA4. PR1000 is an oligonucleotide complementary to positions 980–1000 in the wild-type RmInt1 sequence (454 bp into the IEP sequence) just downstream from the *Eco*RI restriction site. pKGEMA4 was then digested with *Spe*I/*Eco*RI and the fragment obtained was replaced with the corresponding digested PCR product. The sequence of the insert was checked before retrohoming assays.

### Mobility assays

We used a two-plasmid system based on a donor plasmid (pKGEMA4-derived plasmids) encoding IEP followed by ΔORF and a recipient plasmid (pJB0.6LAG) containing a 640 bp fragment of IS*Rm2011-2* including the DNA target site inserted in the lagging strand orientation (Martínez-Abarca et al., 2000). As negative controls, we used a splicing-deficient mutant intron donor plasmid, pKG4dV, and a recipient plasmid lacking the intron insertion site, pJBΔ129. Plasmid pools from *S. meliloti* RMO17 were analyzed by Southern hybridization with probes specific for the intron (ribozyme) and the target DNA (Martínez-Abarca et al., 1998, 2000). Retrohoming efficiency was calculated as a percentage relative to the wild-type intron, by determining the proportion of the recipient plasmids invaded.

### Expression and purification of the MBP-FlagIEP fusion protein in *E. coli*

*E. coli* Rosetta-gami (DE3) pLysS cells were freshly transformed with the pMALFlagIEP expression plasmid or with a mutated form of this plasmid. For starter cultures, a single colony was used to inoculate 5 ml of LB medium containing antibiotics and glucose, and the cultures were incubated overnight at 37°C, with shaking at 270 rpm. An aliquot (1 ml) of the overnight culture was then used to inoculate 50 ml of LB medium + ampicillin + chloramphenicol + glucose, which was incubated at 37°C for 4 h with vigorous shaking, until an OD_600_ of 0.5 was reached. Following IPTG induction, cells were harvested by centrifugation at 5000 × g for 30 min at 4°C, and the pellet was washed twice with cold column buffer (CB: 20 mM Tris-HCl pH 7.4, 200 mM NaCl, 1 mM EDTA, 1 mM DTT, EDTA-free protease inhibitor). The cell pellet was resuspended in 1 ml of ice-cold CB buffer and lysed by three freeze-thaw cycles (alternation between −70°C and +28°C), followed by addition of 7 ml CB. Lysates were subjected to sonication and cleared by centrifugation (16,000 × g for 5 min at 4°C). For protein binding to the column (Econo-Pac Chromatography, Bio-Rad), which was prepared with washed amylose beads (0.5 ml bead volume, New England Biolabs), cleared lysates were incubated for 2 h at 4°C on a rotary shaker. After washing to remove non-specifically bound proteins, MBP-IEP was eluted by adding 2 ml CB supplemented with 10 mM maltose (Sigma-Aldrich). Protein preparations were concentrated and dialyzed with YM-30 centrifugal filters (Amicron Ultra, Millipore) Proteins were analyzed by electrophoresis in Coomassie blue-stained 0.1% SDS-10% polyacrylamide gels and immunoblot analysis with antibodies against the Flag epitope (Sigma-Aldrich). Protein concentrations were determined by the Bradford method, with the Bio-Rad protein assay reagent and BSA as the standard (Bio-Rad).

### *In vitro* transcription and purification

pLMWT was linearized by using digestion with *Nde*I, for the generation of a 908 nt RNA transcript. Unlabeled transcripts were generated with 20 μg of linearized plasmid and 70 units of T7 RNA polymerase in 1 ml reaction buffer containing 1 × transcription buffer (10 × transcription buffer: 150 mM MgCl_2_, 400 mM Tris–HCl, pH 7.5, 20 mM spermidine, and 50 mM DTT), 0.96 mM NTPs, 10 mM DTT and 400 units of RNAseOUT (Invitrogen). Internally labeled RNA transcripts were generated with 4 μg of linearized plasmid, 1 × transcription buffer, 10 mM DTT, 0.96 mM ATP, 0.96 mM CTP, 0.96 mM UTP, 0.064 mM GTP, 50 μCi [α-^32^P]GTP (3000 Ci/mmol; 10 mCi/mL; Perkin Elmer), 80 units of RNAseOUT (Invitrogen) and 2–4 units of T7 RNA polymerase, in a final volume of 50 μl (Chillón et al., 2014). In both cases, RNA synthesis was stopped after 3–5 h at 37°C, by adding an equal volume of gel loading dye [10 M urea, 0.1% (w/v) xylene cyanol and bromophenol blue dyes, 40 mMTris (pH 7.5), 8.3% (w/v) sucrose, and 0.83 mM EDTA]. RNA samples were subjected to electrophoresis in a 5% (w/v) denaturing (7 M urea) polyacrylamide gel. The relevant bands were excised and the RNA molecules were eluted by overnight incubation in elution buffer (0.3 M NaCl, 10 mM MOPS pH 6, 1 mM EDTA). Residual polyacrylamide was removed and the RNA was precipitated with ethanol and glycogen as the carrier. The RNA was dried and dissolved in RNA storage buffer (10 mM MOPS pH 6, 1 mM EDTA).

### Exogenous RT assay

Reverse transcriptase with poly(rA)-oligo(dT)_18_ was carried out as previously described (Muñoz-Adelantado et al., 2003). IEP preparations (5 μM) were assayed in 10 μl of reaction medium (50 mM Tris-HCl pH 7.5, 10 mM KCl, 25 mM MgCl_2_, 5 mM DTT) with 1 μg of poly(rA)-oligo(dT)_18_ or poly(rA), and 2.5 μCi of [α-^32^P]dTTP (800 Ci/mmole; Perkin Elmer). The products (8 μl) were spotted onto Whatman DE81 filters, which were then washed four times with 2 × SSC and counted in a liquid scintillation analyzer (Beckman-Coulter).

### Self-splicing and IEP-assisted splicing

Protein-assisted splicing reactions were carried out with internally ^32^P-labeled RNA and purified MBP-FlagIEP fusion proteins. RNA precursor (1 nM) was incubated in 40 mM Tris-HCl pH 7.5 at 90°C for 1 min, then at 50°C for 1 min and, finally, at room temperature for 1 min. Correct RNA conformation/stabilization was favored by adding 500 mM NH_4_Cl and 5 mM MgCl_2_ to the renatured samples. The splicing reaction was triggered by the incorporation of a 50-fold excess of the MBP-IEP fusion proteins and incubation of the mixtures at 30°C. *In vitro* splicing control reactions from which the protein was omitted were carried out at 50°C for 5 h. Aliquots (1–2 μl) were removed at the indicated timepoints and reactions were stopped by the addition of 10 μl quenching buffer [1.8% sucrose, 1 × TBE, 0.018% xylene cyanol dye, 36% (v/v) formamide, and 25 mM EDTA] and incubation on ice. The products were resolved by electrophoresis in a denaturing 5% polyacrylamide gel. The gel was then dried and the radioactivity quantified with a Personal Molecular Imager FX (Bio-Rad) laser scanning system and Quantity One software (Bio-Rad).

### RNP particle reconstitution

RmInt1-ΔORF RNP particles were reconstituted from RNA synthesized *in vitro* and purified MBP-FlagIEP or the corresponding mutated proteins, with a modified version of a previously described method (Saldanha et al., 1999). Unlabeled precursor intron RNA (2.5 μM) was denatured by heating at 90°C for 1 min and then renatured by successive incubations at 50°C and room temperature for 1 min each in 40 mM Tris-HCl pH 7.5, before the addition of 500 mM NH_4_Cl, 5 mM MgCl_2_, and 5 μM purified MBP-FlagIEP protein. The mixture was placed in a water bath at 30°C for 30 min to 2 h, and the samples were then kept on ice. Freshly prepared reconstituted RNP particles were used for RT and DNA endonuclease assays. Different concentrations of RNA and protein were tested, to optimize the protocol.

### Primer extension

RNP particle preparations were subjected to phenol extraction and the RNA component was precipitated in ice-cold 100% ethanol. Primer extension reactions were carried out essentially as previously described (Molina-Sánchez et al., 2010). RNA samples were resuspended in H_2_O and combined with 5′-labeled P primer (5′-TGAAAGCCG ATCCCGGAG-3′) in 10 mM Pipes (pH 7.5) and 400 mM NaCl. The annealing mixture was heated at 85°C for 5 min, rapidly cooled to 60°C and slowly chilled to 45°C. Extension reactions were initiated by adding AMV RT buffer 1x (5x: 250 mM Tris–HCl pH 8.0, 300 mM NaCl, 50 mM DTT, 30 mM MgOAc), dNTPs, actinomycin D (Sigma-Aldrich), RNase OUT (Invitrogen) and AMV RT (Roche Diagnostics) and incubating at 42°C for 60 min. The reactions were stopped by ethanol precipitation and the products were subjected to electrophoresis in a denaturing 6% polyacrylamide gel. Primer extension products were quantified with Quantity One software (Bio-Rad) and excision efficiency was determined as 100 × [Lariat/(Lariat + Precursor)].

### DNA cleavage/reverse splicing activity

DNA cleavage assays were carried out on a 70-mer single-stranded DNA oligonucleotide containing the intron insertion site at position 35, which was 5′ end-labeled with [γ-^32^P]ATP (6000 Ci/mmol; Perkin-Elmer) and T4 polynucleotide kinase (New England Biolabs), or 3′ end-labeled with terminal transferase (New England Biolabs) and cordycepin 5′-triphosphate [α-^32^P] 3′-deoxyadenosine (6000 Ci/mmol; Perkin Elmer). DNA substrates were gel-purified and eluted from acrylamide, essentially as previously described (Molina-Sánchez et al., 2010). DNA oligonucleotide (300,000 cpm) was incubated with reconstituted RNP particles (2.5 μl; ~900 nM) at 37°C for 30 min to 2 h in reaction buffer (50 mM Tris-HCl pH 7.5, 10 mM KCl, 25 mM MgCl_2_, 5 mM DTT). The reactions were cleaned by extraction with phenol-chloroform-isoamyl alcohol (25:24:1) followed by ethanol precipitation, and the products were analyzed by electrophoresis in denaturing 7 M urea-6% (w/v) polyacrylamide gels. The gels were scanned and the products were quantified with Quantity One software (Bio-Rad), with the results expressed as a percentage of the activity of RNP complexes reconstituted with the wild-type MBP-FlagIEP fusion protein.

## Results and discussion

### *In vivo* functionality of the MBP-FlagIEP

**Figure 1:**
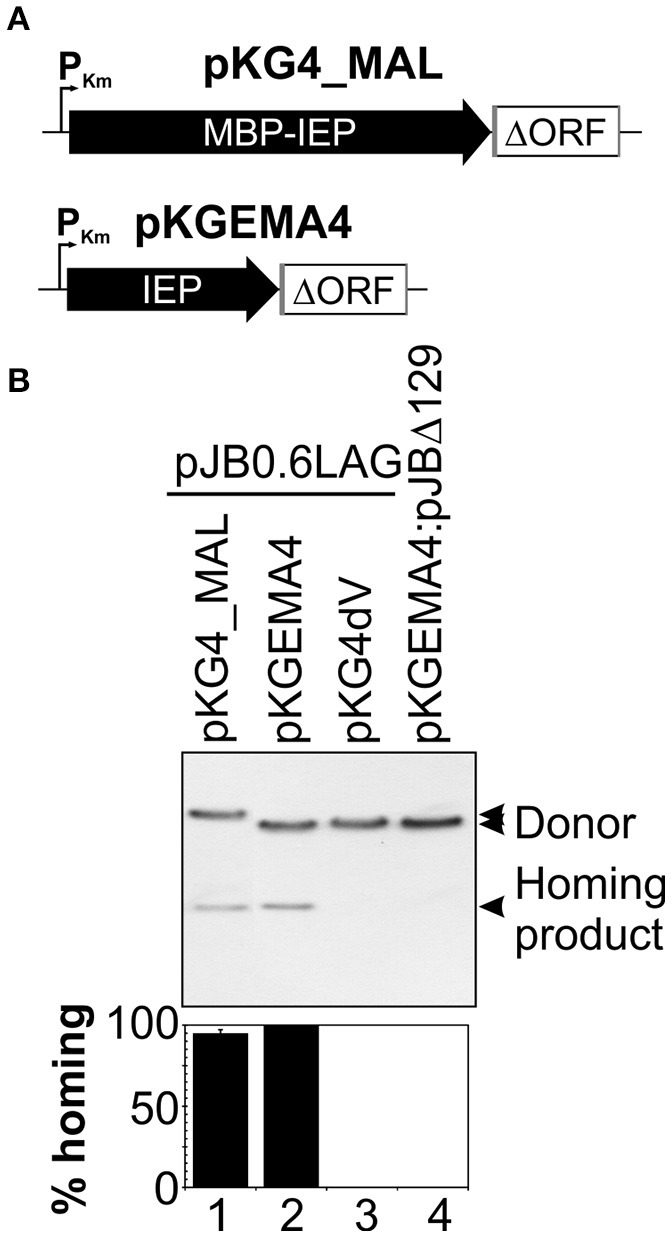
**The MBP-FlagIEP fusion protein is functional *in vivo***. **(A)** The scaled scheme shows the constructs used as intron donors. pKG4_MAL is a derivative of pKGEMA4 in which the IEP has been replaced by the fusion protein MBP-FlagIEP. P_Km_, kanamycin promoter; black arrows identify the IEP; an open box flanked by gray lines corresponds to the intron ΔORF and short −20/+5 exons, respectively. **(B)** A homing assay is shown in the upper panel. The donor constructs indicated above were used to transform *S. meliloti* RMO17, an intron-less strain, containing the recipient plasmid pJB0.6LAG. The plasmid pools were analyzed by Southern hybridization with a DNA intron probe. An intron donor plasmid harboring a mutation in the catalytic domain of the ribozyme (pKG4dV) or a recipient plasmid lacking the recognition DNA target region (pJBΔ129) was used as a control. The lower panel corresponds to quantification of the retrohoming efficiency expressed as a percentage relative to that for the pKGEMA4 construct. The data shown are the means for at least three independent colonies ± standard errors.

The IEPs of group II introns are proteins with a high proportion of positively charged amino acids and an alkaline isoelectric point, and their stability and solubility are, therefore, usually low (Mohr et al., 2013; Zhao and Pyle, 2016). We overcame these problems by producing the RmInt1 IEP as a fusion with the maltose binding protein (pMAL, New England Biolabs). The construct encoded the maltose binding protein (*mal*E, MBP) followed by the Flag epitope, all in-frame with the N-terminal region of the RmInt1 intron ORF. We assessed the competence of the MBP-FlagIEP fusion protein for assisting the *in vivo* activities of RmInt1 intron RNA, by carrying out retrohoming assays with a two-plasmid (donor/recipient) system (Martínez-Abarca et al., 2004). We used an intron donor plasmid derived from pKGEMA4 (Nisa-Martínez et al., 2007) and encoding the fusion protein under the control of the kanamycin promoter (P_Km_) and upstream from the RmInt1 ΔORF ribozyme flanked by exon sequences −20/+5 (Figure 1A). *S. meliloti* RMO17, an intronless strain, harboring the recipient plasmid pJB0.6LAG was transformed with the pKG4_MALFlagIEP intron donor plasmid (Figure 1B, lane 1). Homing efficiency was slightly lower (21%) than for the wild-type protein construct, pKGEMA4 (lane 2). The MBP-IEP fusion protein therefore seemed to be fully functional *in vivo*.

### Production, purification, and RT activity of the RmInt1 IEP

The MBP-FlagIEP cassette was inserted downstream from the *tac* promoter and the fusion protein was produced in Rosetta-Gami (DE3) pLysS cells after IPTG induction. The protein was purified further by affinity chromatography on amylose resin (see Materials and Methods). The one-step purification procedure yielded large amounts of soluble protein (1–60 mg/L of culture), but the preparation was heterogeneous, containing protein aggregates and partially degraded/synthesized protein fragments (not shown). Quantification of the various bands suggested that the full-length protein preparation was about 70% pure (checked for several different preparations).

**Figure 2:**
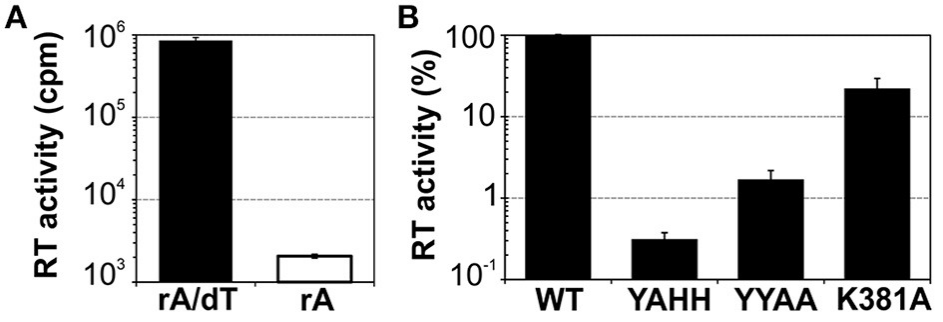
**Purified MBP-FlagIEP has reverse transcriptase activity *in vitro***. **(A)** The bar graphs show the reverse transcriptase activity of the MBP-FlagIEP fusion assayed with poly(rA)/oligo(dT)_18_ (black bar), or with poly(rA) [in the absence of oligo(dT)_18_, white bar]. The RT activity is expressed in counts per minute (cpm). The data shown are the means for at least three independent assays and three to seven independent MBP-FlagIEP preparations. Error bars represent the standard errors. **(B)** The RT activity of several mutant proteins was evaluated relative to the wild-type fusion protein. Note that the Y axis is represented in logarithmic scale. RT values were derived from at least three experimental replicates, with three different protein preparations used for each mutant. Error bars correspond to the standard errors.

We assessed the functionality of the protein, by carrying out reverse transcriptase assays with the exogenous substrate poly(rA)/oligo(dT)_18_ (Figure 2A). The MBP-FlagIEP fusion protein displayed significant RT activity in the presence of the RNA substrate and the DNA primer (8.6 × 10^5^ cpm), whereas the absence of oligo(dT)_18_ resulted in much lower levels of cDNA synthesis (2 × 10^3^ cpm). As a control, we also investigated three other fusion proteins in which conserved residues of the RT and maturase domains had been mutated (Figure 2B). As expected, cDNA synthesis was abolished in the RT-deficient YAHH mutant (0.3% wild-type), in which two essential aspartate residues in RT domain 5 were replaced with histidines (Muñoz-Adelantado et al., 2003). The YYAA maturase-null mutant, in which two conserved tyrosine residues at positions 354–355 in the RGWXNYY maturase motif were replaced with two alanine residues, had very low levels of RT activity (1.7% wild-type). In addition, the K381A mutant, with a substitution of the lysine 381 residue in the conserved maturase R(K/R)XK motif decreasing intron excision rates to 30% those for the wild type (Molina-Sánchez et al., 2006, 2010), had low levels of RT activity (22% wild-type). These results suggest that these residues might be relevant for the substrate docking required for cDNA synthesis, to somewhat different degrees. Thus, MBP-FlagIEP was stable and active when assayed alone in the absence of the intron RNA.

### RmInt1 splicing *in vitro*

**Figure 3:**
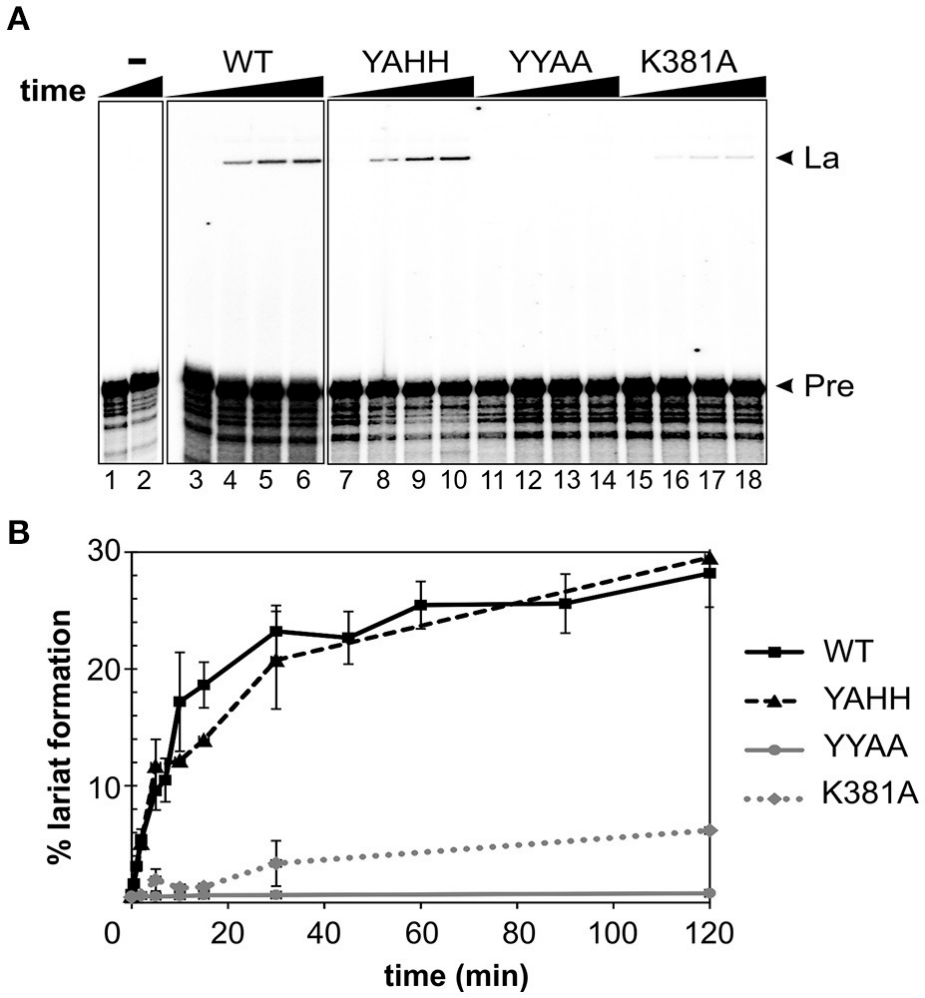
**RmInt1 splicing efficiency *in vitro*. (A)** The left panel (lanes 1–2) shows the inability of RmInt1 to self-splice *in vitro*. Splicing reactions were carried out by incubating ^32^P-labeled, 905 nt RmInt1 ΔORF RNA with 5 mM MgCl_2_ for 0 and 300 min. The panels on the right (lanes 3–18) show RmInt1 IEP-assisted splicing, as evaluated by incubating a ^32^P-labeled ΔORF RNA precursor with different protein preparations in a buffer containing 5 mM MgCl_2_ for various times. The radioactively labeled precursor ΔORF RNA was incubated with a 50-fold excess of wild- type MBP-FlagIEP fusion protein (middle panel) or RmInt1 IEP mutant proteins (right panel) for 0, 5, 30, and 120 min. The arrows on the right indicate the products obtained: La, fully spliced lariat RNA; and, Pre, precursor RmInt1 ΔORF RNA. **(B)** Kinetic data, including additional time points, are plotted as the percentage lariat formation relative to the level of precursor RNA. Lines in black/squares represent the MBP-FlagIEP wild-type fusion protein; the RT-deficient mutant (MBP-FlagYAHH) is indicated by a broken black line/triangles; maturase domain mutants are indicated with a solid gray line/circles (MBP-FlagA354A355) or a broken line/diamonds (MBP-FlagA381). The experiment was repeated twice, with at least two independent purified fusion proteins for each mutant.

As the fusion protein itself has RT activity, we investigated whether this protein could interact successfully with the intron ribozyme. Previous studies performed by our group revealed that RmInt1 efficiently self-spliced *in vitro* in the presence of high-magnesium (100 mM) buffer, but that these conditions gave rise to multiple products corresponding in size to the lariat intron-3′exon intermediate, and lariat or circle (double band) introns (Costa et al., 2006; Chillón et al., 2014). IEP-promoted splicing of a 906 nt ΔORF precursor RNA was monitored over time (Figure 3). Protein-assisted splicing of the ΔORF RNA precursor resulted in a single lariat RNA product (Figure 3A). A two-exponential model fitted the data well, with an initial fast reaction (>60% of lariat formation was completed in the first 15 min) followed by a phase of slow reactivity (Figure 3B). In the presence of MBP-FlagIEP wild-type, the percentage lariat formation reached 31%. IEP-promoted *in vitro* splicing was performed in different Mg^2+^ concentrations, but the results were not improved by increasing MgCl_2_ concentration beyond 5 mM (not shown). As previously reported, this low-magnesium buffer does not allow intron excision in the absence of the IEP (Figure 3, left panel). Hence, IEP assistance reduces magnesium requirements and accelerates lariat formation, preventing the production of secondary products. A two-phase reaction has also been reported for LtrA-assisted splicing, with a fast phase in which about 50–75% of the molecules react, followed by a slow reaction until the fraction spliced finally reaches 75–95%, depending on the conditions (Wank et al., 1999; Matsuura et al., 2001; Noah and Lambowitz, 2003; Cui et al., 2004).

We also evaluated the ability of mutant proteins to assist the splicing of the intron RNA. Consistent with our *in vivo* results (Molina-Sánchez et al., 2010), there was no significant difference in lariat formation between the YAHH mutant and the wild-type MBP-FlagIEP (Figure 3). Likewise, the splicing-deficient YYAA mutant was unable to induce intron excision *in vitro*, and substitution of the K381 residue greatly decreased splicing efficiency (20% wild-type levels). Our results therefore indicate that the catalytic residues for RT are not directly involved in RmInt1 splicing, and are consistent with a possible alteration of the domain X mutant IEPs protein structure and binding properties.

Together, these results indicate that the MBP-FlagIEP fusion protein functionally complements the ribozyme activity of the RmInt1 intron and is therefore suitable for use in RNP particle reconstitution *in vitro*.

### Reconstituted RNP particles have DNA endonuclease activity

**Figure 4:**
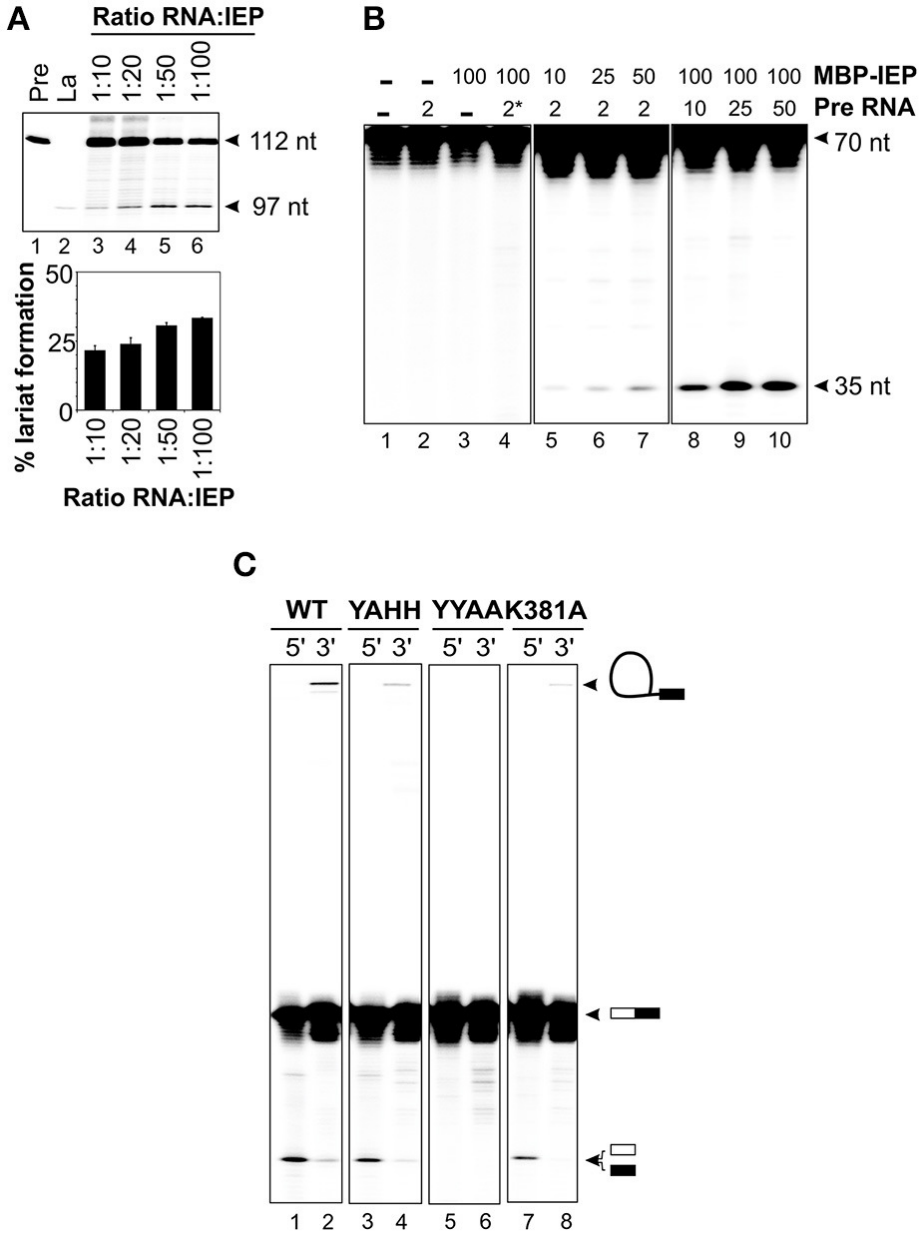
**Reconstitution of RNP particles with the MBP-FlagIEP fusion protein and *in vitro*-synthesized intron RNA. (A)** Lariat formation during the RmInt1 RNP particles reconstitution process was estimated by primer extension. RNP particles reconstituted with different ratios of ΔORF RNA precursor and MBP-FlagIEP fusion protein were subjected to phenol extraction and used as the template for reverse transcription reactions with an intron-specific primer. The intron lariat gave rise to a 97 nt cDNA, whereas the RNA precursor generated a 112 nt product. *In vitro*-transcribed RNAs corresponding to the precursor and lariat RNA molecules were used as controls. The percentage lariat formation is plotted below. Results were verified by analyses of at least three different IEP preparations and are presented as the mean ± standard error. **(B)** DNA endonuclease activity on ^32^P-labeled single stranded DNA substrates (70 nt). DNA cleavage activity resulted in a 35 nt product. The control samples are shown in the left panel: neither RNA nor MBP-FlagIEP, only RNA precursor, only MBP-FlagIEP, and RNA precursor with a domain V mutation (^*^) plus MBP-FlagIEP. In the middle panel, the DNA substrate was incubated with RNP particles reconstituted from a constant amount of RNA precursor (2 pmol) and increasing amounts of MBP-FlagIEP (10, 25, and 50 pmol). In the right panel, the DNA substrate was incubated with RNP particles reconstituted from a fixed amount of MBP-FlagIEP (100 pmol) and increasing amounts of ΔORF RNA precursor incubated with the ssDNA target substrate. **(C)** Cleavage efficiency for 70 nt single-stranded DNA substrates ^32^P-labeled at the 5′ and 3′ ends, with RNP particles reconstituted from 5 μM wild-type to mutant IEPs and 2.5 μM ΔORF precursor RNA, determined after 2 h of incubation. The molecules resulting from the DNA endonuclease reaction are indicated on the right (not drawn to scale): The white box represents the 5′ exon and the black rectangle corresponds to the 3′ exon; the black line identifies the intron RNA.

We investigated whether the IEP and the excised lariat RNA were sufficient for RmInt1 DNA endonuclease activity, by reconstituting RNP particles from the components purified *in vitro*, as previously described by Saldanha et al. (1999). The ΔORF precursor RNA was incubated with MBP-FlagIEP, in different ratios, at 30°C for 30–60 min. These experiments assessed the ability of the protein to promote RmInt1 RNA splicing, assuming that the complex remained intact to generate active RNP particles containing the protein and the intron lariat. We tested this hypothesis, by evaluating lariat formation by primer extension (Figure 4A). The cDNA was synthesized with an intron-specific primer complementary to a sequence located 80–97 nucleotides from the 5′ end of the intron (Muñoz-Adelantado et al., 2003). The unspliced precursor-derived products were detected as a 112 nt band, whereas the cDNA corresponding to the lariat molecules migrated at 97 nt. As expected, the amount of lariat formed increased with the amount of MBP-FlagIEP added, before reaching a plateau at about 30% lariat (ratio RNA:IEP 1:50), with no further increase even if the amount of protein was doubled. This finding is consistent with those for our IEP-assisted splicing experiments (Figure 3), suggesting that considerable large proportion of intron RNA cannot take part in the reaction.

We assessed the best conditions for RNP particle reconstitution, by carrying out DNA endonuclease assays with titrated reconstitution reactions (Figure 4B). In DNA endonuclease experiments, we incubated reconstituted RNP particles with a 5′ end-labeled 70 nt single-stranded DNA substrate containing the intron recognition site located at position 35. We found that adding the intron RNA or the fusion protein alone did not generate a cleavage band (lanes 2 and 3). Incubation of the substrate with RNP particles reconstituted with a fixed amount of RNA (2 pmol) and increasing amounts of MBP-FlagIEP revealed that cleavage efficiency increased with the amount of protein used for reconstitution, probably reflecting the active RNP complex levels (lanes 5–7). Likewise, for RNP particles reconstituted with a constant amount of MBP-FlagIEP (100 pmol) but increasing amounts of precursor RNA, cleavage product levels increased with the amount of precursor RNA, reflecting higher levels of active RNP particle formation (lanes 8–10). No DNA endonuclease activity was detected when the RNP particles were reconstituted with a precursor RNA with a mutated domain V (GUU → CGA) resulting in a loss of splicing ability (lane 4). Suitable conditions for RNP particle reconstitution would therefore be a 1:2 ratio of RNA to IEP and 2.5 μM intron RNA. Higher concentrations of RNA did not increase the efficiency of the reaction, with DNA endonuclease activity eventually becoming saturated. These data are consistent with previous findings for LtrA, for which kinetic analyses of splicing and RNA binding reactions showed that the LtrA protein bound to Ll.LtrB RNA as a dimer (Saldanha et al., 1999; Wank et al., 1999; Rambo and Doudna, 2004). However, this interpretation should be viewed with caution, because it conflicts with findings for RNP complex stoichiometry based on recent structural determinations (Gupta et al., 2014; Qu et al., 2016; Zhao and Pyle, 2016).

Having established appropriate reaction conditions, we investigated whether the RNP particles reconstituted with different mutant proteins were active in DNA cleavage. The wild-type fusion protein cleaved the substrate, generating a 35 nt product corresponding to the intron insertion site that was more clearly visible when the substrate was labeled at the 5′ end (Figure 4C, lane 1) than when it was labeled at the 3′ end (lane 2). The presence of high-molecular weight bands in lanes containing 5′ end-labeled substrates would correspond to full reverse-splicing products. On the contrary, 3′ end-labeled substrates might reveal both full and partial reverse splicing. We observed partial reverse splicing products with wild-type reconstituted RNP particles (lane 2), but we cannot rule out that full reverse splicing occurs. These data correlate with those obtained for other group II introns, that shown significantly more partial reverse splicing than full reverse splicing (Matsuura et al., 1997; Saldanha et al., 1999; Aizawa et al., 2003). Similar results were obtained with RNP complexes reconstituted with the MBP-YAHH RT-deficient mutant, which displayed high levels of DNA cleavage activity (90–95% of wild-type, lane 3). By contrast, as expected reconstituted RNP particles using the MBP-YYAA mutant protein were unable to cleave the DNA substrate at the insertion site, resulting in the absence of reverse splicing bands (lanes 5–6). Finally, RNP particles reconstituted with the K381A mutant protein had lower levels of cleavage activity than wild-type complexes (78%; lane 7), consistent with the lower RT and splicing activities observed, and only bands generated by partial reverse splicing were also detected (lane 8). These findings may explain why this mutant has no retrohoming activity *in vivo* (Molina-Sánchez et al., 2010).

## Concluding remarks

In this work, we developed a protocol for producing active RmInt1 IEP. The MBP-Flag IEP fusion protein was competent for reverse transcription and facilitated intron splicing *in vitro*, reducing Mg^2+^ requirements. Like other group II introns, RmInt1 IEP-dependent splicing occurred in a two-phase reaction: most of the lariat molecules were generated rapidly, early in the incubation period, and there was then a slow reaction phase. We were also able to produce RNP particles *in vitro* from the two purified components, and these complexes efficiently cleaved single-stranded DNA substrates, with the intron RNA mostly remaining linked to the 3′ exon. We also obtained control RNP particles, which reproduced *in vitro* the activities previously described for RNP-enriched fractions recovered *in vivo* (Molina-Sánchez et al., 2010).

RNP particles formed *in vivo* have already been purified with tagging and other similar procedures (Zerbato et al., 2013; Qu et al., 2016), but, to our knowledge, there have been only two reports of active RNP complex reconstitution *in vitro* from purified components (Ll.ltrB, Saldanha et al., 1999 and subsequent studies arising from this work; and, *B.h.*I1, Robart et al., 2007). Further studies increasing our insight, the efficiency of RNP particle reconstitution and the purity of RNP particle preparations will provide tools for exploring new biotechnological applications for group II introns lacking the DNA endonuclease domain.

## Author contributions

NT designed the project; MM purified and reconstituted the RNP complex and performed activity assays; FG conceived and constructed the pMAL_IEP purification system; NT, FG, and MM discussed the results and wrote the paper. All the authors approved the final version of the manuscript.

## Funding

This work was supported by research grant BIO2014-51953-P of the *Plan Nacional de I*+*D*+*i*, Biotechnology program from the Spanish *Ministerio de Economía y Competitividad* including ERDF (European Regional Development Funds).

### Conflict of interest statement

The authors declare that the research was conducted in the absence of any commercial or financial relationships that could be construed as a potential conflict of interest.
